# Social connection, relationships and older lesbian and gay people^1^

**DOI:** 10.1080/14681994.2014.963983

**Published:** 2014-10-31

**Authors:** Catherine Barrett, Carolyn Whyte, Jude Comfort, Anthony Lyons, Pauline Crameri

**Affiliations:** ^a^ Australian Research Centre in Sex, Health and Society, La Trobe University, Melbourne, Australia; ^b^ School of Public Health, Faculty of Health Sciences, West Australian Centre for Health Promotion Research, Curtin University, Perth, Australia

**Keywords:** gay, lesbian, relationships, homophobia, social connections

## Abstract

This paper presents data from a small study exploring the impacts of homophobia on the lives of older lesbian and gay Australians. Eleven in-depth interviews were conducted with older lesbians (6) and gay men (5) ranging in age from 65 to 79 years. The study found that participants’ sense of self was shaped by the dominant medical, legal and religious institutions of their youth that defined them as sick, immoral or criminal. Participants described enforced “cure” therapies, being imprisoned, having employment terminated and being disowned and disinherited by family. In this context, intimate relationships and social networks provided refuge where trust was rebuilt and sexuality affirmed. Many created safe spaces for themselves. This equilibrium was threatened with increasing age, disability and the reliance on health and social services. Participants feared a return to institutional control and a need to “straighten up” or hide their sexuality. In response, partners stepped into the role of caregiver, at times beyond their capacity and at a cost to their relationship. The study describes the importance of understanding social connections in the lives of older lesbians and gay men. It highlights the need for inclusive services to ensure that social networks are supported and that health and well-being are promoted.

## Introduction

Many older lesbians and gay men have lived through a time when their only protection against heterosexist violence and discrimination was to make themselves invisible, to publicly deny their sexual orientation and pass as heterosexuals (Barrett, 2008). Disclosure could result in imprisonment, enforced medical interventions and “cures,” or the loss of employment, family and friends (Barrett, 2008; Leonard, Duncan, & Barrett, 2012). Prior to the gay liberation movement, the experience of coming out or disclosing their sexual orientation had a detrimental impact on their sense of self, relationships and social connections. Older lesbians and gay men are less likely than their post-liberation peers to have had their sexual orientation affirmed or celebrated, are less likely to have developed positive self-image and self-esteem (Cronin & King, 2014) and more likely to have experienced fracturing of significant relationships.

The discrimination experienced by older lesbians and gay men has also affected their health adversely and their willingness to access health services (Fredriksen-Goldsen et al., 2011). They have higher rates of disability, depression and loneliness than their heterosexual counterparts (Fredriksen-Goldsen et al., 2011). They have fewer social networks, are more likely to be single and live alone (Guasp, 2011) and are at greater risk of social isolation (Fredriksen-Goldsen et al., 2011). Friendships are integral to building social networks and have been shown to be a key factor in positive mental health (Lyons, Pitts, & Grierson, 2013). However, many older lesbians and gay men report that their sexuality is a barrier to building friendships in the broader community (Cronin & King, 2014). The social isolation this creates further diminishes their health and well-being (Fredriksen-Goldsen et al., 2011).

Despite these disparities, older lesbians and gay men are less likely to access services they feel are needed, because they fear discrimination (Guasp, 2011). They are also more likely to be reliant on partners and friends for support as they age (Fredriksen-Goldsen et al., 2011), which is attributable in part to the fact that they are less likely to have children and less likely to have regular contact with their biological families (Guasp, 2011).

To address these disparities, service providers must understand the historical experiences and needs of older lesbians and gay men (Barrett, Turner, & Leonard, 2013). However, until recently, older lesbians and gay men were almost completely ignored in gerontology, policy development and legal reform (Harrison, 2006). This created a cycle of invisibility (Harrison, 2001). Sexuality was hidden because it was not safe – fostering an illusion that reforms were not required. In turn, the absence of reforms meant that older lesbians and gay men continued to believe it was unsafe to disclose their sexual orientation. Research demonstrates that many service providers do not believe that they need to be inclusive because it does not occur to them that they have any lesbian or gay clients (Barrett, Harrison, & Kent, 2009; GLBTI Retirement Association Inc. [GRAI], 2010). There is also a commonly held myth that older people are asexual and therefore cannot be sexually diverse (Barrett, 2011). The failure to address the needs of older lesbians and gay men is an indirect form of discrimination (Hughes, 2006, 2007) that is currently being addressed in a number of countries around the world.

This paper describes a small ethnographic study documenting the effects of discrimination on older lesbians and gay men in Australia. The project was funded in response to a growing body of research, linking higher rates of depression and anxiety to the experiences of homophobic discrimination (Corboz et al., 2008). Funding was provided by *beyondblue*, a national organisation working to address issues associated with depression, anxiety and related disorders in Australia. The study was conducted by Val's Café at the Australian Research Centre in Sex, Health and Society at La Trobe University, in collaboration with the Western Australian Centre for Health Promotion Research at Curtin University between 2012 and 2013. The study documented the effects of discrimination on relationships and social connections and highlighted the need for improved services and further research.

## Aims of the research

The aims of the research were to document older lesbian, gay, bisexual, transgender and intersex people's experiences of discrimination, particularly with regard to its impact on their relationships with partners, friends and family, and to understand how these experiences are implicated in higher than average rates of depression and anxiety. The study was largely exploratory. We did not specify hypotheses. However, we expected that accounts from participants would focus strongly on the impact of discrimination on their relationships and psychological well-being given the growing body of research, linking higher rates of depression, anxiety and related disorders among lesbians, gay, bisexual, transgender and intersex (LGBTI) Australians to their experiences of homophobic and transphobic discrimination (Corboz et al., 2008), as well as evidence for a strong protective effect of social support (Lyons et al., 2013).

## Method

### Study design

In-depth interviews were conducted to explore participants’ experiences of discrimination, the effects of discrimination, the experience of anxiety and depression and whether participants felt that there was a connection between discrimination and their depression or anxiety. Ethics approval for the project was granted by the La Trobe University Human Research Ethics Committee.

### Procedure for recruitment

A recruitment flyer was developed and circulated to LGBTI groups and service providers in Victoria, Western Australia and Queensland. The study was restricted to three Australian states because of the limited resources and small scale of the study. The flyer clarified that the researchers were seeking to interview LGBTI people aged 65 years or more who had experienced homophobic or transphobic discrimination and depression or anxiety.

### Participants

Twelve interviews were conducted with participants from Victoria (*n* = 5), Western Australia (*n* = 6) and Queensland (*n* = 1). Six participants identified as lesbian/gay women, five as gay men and one as transgender. No intersex or bisexual people were recruited. This paper focuses on interview data from the 11 lesbians and gay men who participated. The age range of participants was 65–79 years with a mean age of 70 years (SD = 4.7 years).

### Procedure

Nine interviews were conducted face-to-face in participants’ homes. Two of these were conducted in nursing homes. Further three interviews were conducted by phone. Each interview lasted between 40 and 90 minutes. Participants were asked about their experiences of homophobic discrimination and the effects on their lives, particularly in relation to depression and anxiety. They were invited to share an image to help complement their spoken account and explore experiences that they might find difficulty to talk about (Eisner, 2008; Frawley, Barrett, & Dyson, 2013). Participants were invited to select an image that would not identify them and, in the case of phone interviews, to email or post the image to the researcher. Given the sensitive and potentially distressing nature of the experiences that were being recounted, participants were provided with details of professional support services and invited to identify strategies for self-support before the interview was conducted.

The interviews were audio recorded and transcribed using a professional transcription service approved by La Trobe University Human Research Ethics Committee. The draft narratives and full transcripts were returned to participants for verification and to provide participants with the opportunity to make changes to protect their identity. Feedback from participants was considered important to maximise the report's credibility (Patton, 2002) and authenticity (Winter, 2002). It was also considered important to maximise participants’ control over how their lives were represented given the historical pathologising of lesbian and gay sexuality. To ensure anonymity, all participants’ names were replaced with pseudonyms when reporting findings in this article.

### Data analysis

The interview transcripts were analysed thematically adapting Ritchie and Spencer's “framework” to identify common themes and significant differences (Ritchie & Spencer, 1994). Analysis was independently undertaken by two of the authors of this article and then discussed with the broader team to identify key themes. In addition, individual narratives were constructed from the transcripts. This involved ordering events into a chronological sequence, editing some data and providing thematic headings within each narrative.

## Findings

Many of the participants’ responses in interviews involved recounting the devastating impacts of homophobia on their lives, their health and their relationships. Narratives about discrimination and its effects included strong themes around the effects of discrimination on relationships and social connections. Three major themes were identified, each of which revolved around key relationships: early relationships with biological family; intimate relationships; and friendships and the broader community. A fourth theme was further identified, which centred on ageing, disability and support services and how these changed relationships and social networks.

### Early relationships with biological family

Participants described the heartache of rejection by their biological families and the consequences on their lives. Pam (not her real name) reported that her family gave her “the flick” after she came out; adding that her sister-in-law “could not even bear to be in the same room … I was persona non grata.” In response, Pam recalled, I had to “distance myself from my family completely” in order to maintain her mental health. Relationships with family were often irreparably damaged. Gerri recounted, my mother said I was “in the gutter and that's where I'd stay while I chose to live this life. … It's not a good feeling when you are told that by your mother. I'll never forgive mum for that.”

While some participants severed contact with family, others tried to build rapport in the hope that their sexual orientation would be accepted over time. Larry described being placed in a psychiatric institution and given shock therapy at the age of 14 after his parents became aware that he was gay. He later joined the army to get away from his father, but was discharged after disclosing to the army chaplain that he was gay. Larry felt that his mother embraced his sexuality but his father continued to reject him and later disinherited him.

Rejection by family was also experienced by Dawn when she left her husband and children to be in a lesbian relationship. Dawn felt pressured to leave her hometown and to withdraw contact from her children because her family believed that the children “would become … corrupted by our relationship … that I would corrupt the children and they would be[come] deviants or something.” Dawn missed key milestones in her children's lives, including her daughter's wedding and was not reconciled with her son until 30 years later. She listed the losses she experienced: “I had a loss of identity for a start, a loss of children, a loss of home, a loss of income, a loss of parenting, a loss of friends.” Dawn's parents removed her images from family photo albums and she described how “the rejection by so many people really broke my spirit.” In the immediate period after leaving her husband, she took an overdose of sleeping pills in an attempt to kill herself.

These early experiences of rejection by biological family members shaped participants’ sense of self, social connection and future relationships. The impacts of this lack of family support were noted by Patrice who said, “the bottom line is [if] you get support first off from your family it does make things a little easier for you … it gives you that extra support, that extra confidence, you know.” In narratives about family support, participants described their appreciation for even small gestures of recognition, particularly in their teenage years when they were still dependent on family.

The importance of family support was highlighted by participants who described significant efforts to regain family support. They also demonstrated a great capacity to forgive family members when they were rejected. For example, Dawn assisted her siblings to care for her father after her mother died and he was diagnosed with Alzheimer's disease. During this time her father apologised and Dawn noted, “he was instantly forgiven.” Expectations of family were lowered; participants did not expect unconditional love and often worked hard at getting families on their side.

Rejection by biological family has significant effects on the lives of older lesbians and gay men (Barrett, 2008; Leonard et al., 2012). As a result, they missed the opportunities for affirming and celebrating sexual orientation (Cronin & King, 2014) and this had enduring effects. For example, Larry described how the view of homosexuality as deviant or disordered “burns its little tentacles into your brain and it stays there, believe me it stays there.”

### Intimate relationships

A lack of affirmation from family, and society more broadly, appeared to influence perceptions and experiences of intimate relationships in later life. Intimate relationships were described as the first and sometimes the only place where sexual orientation was affirmed and valued. Larry recalled how he believed his sexuality was “a sickness” until he met his first partner who affirmed his sexuality by saying there was “nothing wrong with me.” Intimate relationships also provided a safe space, free from discrimination. In reflection on this Pam noted, “the effect that [discrimination] has [is that] there is **you** and the **rest of them** and you are trying to make your own little space.” Similarly, Gerri said: “you feel like you've come home. … this is what I want, I'm safe here and love grows, it definitely grows.” In these ways, intimate relationships appeared to help heal the rejection of biological families (Smalley, 1987) and at times provided the only place where it was safe to be gay or lesbian.

In the longer term, intimate relationships provided an important source of psychological support to manage the everyday experience of homophobia. Cliff reflected, “sometimes it's my partner who understands what I might be going through … a person who's experienced something can certainly relate it much better.”

It may not be surprising then that intimate partners were greatly valued and often referred to as “soul mates.” They provided a significant mediating factor against marginality (Cronin & King, 2014; Heaphy, 2009) that enabled many older lesbians and gay men to survive extraordinary experiences of discrimination. As other studies show, the positive effects of such relationships for lesbians and gay men tend to be sustained over time with those in long-term relationships having more positive attitudes towards ageing than those who are single (Heaphy, 2009).

Although intimate relationships were an important source of social connection, being in a relationship also meant that sexual orientation was more visible, increasing the likelihood of rejection and discrimination. Pam recounted that when she disclosed her sexuality, “my family didn't want to have anything to do with me … because I had a relationship it made it so much worse. If I wasn't in a relationship they could have glossed over it.” The responses of family and the broader community meant that some participants felt the need to hide their relationships to be safe.

The pressure to hide relationships to avoid discrimination created tension (Smalley, 1987) and social isolation. Gerri described declining an invitation to her niece's wedding because she was not sure how people would respond to her partner. She reflected that not being able to share her partner “pisses you off. It does make you angry and sad at the same time and I think if you let yourself dwell on it too much it can be very hard.” The consequences of this for Gerri were that she felt, “boxed in, you can't open up. It is very sad that you have to hide when it's so natural to you and the horrible things that people say and do because you are not like them.”

The decision to straighten up or hide a relationship was at times a point of dissonance in relationships. Larry described how his partner was “ashamed of being found out he was a homosexual so … I could not tell anybody I was a homosexual because I didn't want to hurt him.” While Larry understood his partner's fear, he lamented the lack of recognition of his own sexuality adding “I have lived all my life wanting to be recognised.”

Having an intimate partner also changed the interaction that some participants had with service providers. When Pam's partner became ill, they moved to a rural area to reduce costs and Pam became her partner's full-time caregiver. Pam described how her partner “wanted to jump right into the closet again to the point of telling me off that we shouldn't do things like holding hands. I had to mind ‘p's and ‘q's and not be so obvious.” As others have found (Jordan & Deluty, 2000), having to hide their sexual orientation again after so many years led to dissatisfaction in some relationships.

The hidden nature of some intimate relationships created difficulties around the death of a partner. Gerri described her family's surprise at the extent of her grief when her partner died, believing that she and her partner were just two women “living together and sharing expenses.” Gerri experienced significant difficulty at work because she had not disclosed the nature of her relationship, describing how it was “hell at work … I had to hold [the grief] in.” The lack of relationship recognition resulted in disenfranchised grief (Westwood, 2013), with the loss not being openly mourned, supported or acknowledged (Doka, 1989). For some other participants, the funeral of a partner was the first time their relationship received recognition. After decades of rejection by family, Larry described that at his partner's funeral “… there was all my family, nephews, nieces, all our friends and there must have been about 200 people in there and I send him out with Shirley Bassey singing Hey Big Spender…[he] didn't go out alone.”

### Friendships and the broader community

A broad range of friendships and social groups provided important support networks and a sense of social connection. Tim reflected that he joined a gay social group that “provides friendship … you know it's doing us a service, that's something we require, you can't just sort of isolate yourself.” Similarly, Cliff noted that friendships were important to “help you through” difficult times.

The capacity to embrace friendships and the characteristics of friends were debated. Amanda suggested that some lesbians enter relationships thinking, “okay I am safe here, let's put up the wall,” having little engagement with the broader heterosexual community. Amanda reported that this often led to a “very claustrophobic relationship where you start to rely on each other just too much. … You just cannot get everything you want and need in life from one person.” Amanda believed that “in relationships you should get out and grow. You don't want to become inward looking because you just won't survive.” In her own life, Amanda engaged a broad range of people beyond her circle of lesbian friends. She reported that lesbians who did not do this were at risk of depression if their relationships ended because “there is nothing else … working” in their life.

Although Amanda engaged successfully with the broader community, others had more mixed experiences of heterosexual friendships. Noel was imprisoned at the age of 17 for the “abominable crime of buggery” and described losing heterosexual friends who were concerned that they could be seen as “consorting with a known criminal.” Larry described his close friendship with a heterosexual couple that was severed after the couple became pregnant and did not want gay men near their child. The potential homophobic tension in relationships was highlighted by Gerri who described that by coming out, you “know who your true friends are … some of them think it's a disease and they might catch it, so you don't hear from them, they disappear.”

For participants in this study, heterosexuality was not an exclusion criterion in relationships; rather it was the capacity to value and affirm sexual orientation that determined whether or not friendships were formed and sustained. This phenomenon has been described elsewhere by older lesbians and gay men as “My People” or social networks of supporters who value and affirm sexual diversity (Barrett, 2008). These friendship networks may include ex-lovers (Cook-Daniels, 1997) and may be afforded the status of family (Guasp, 2011) sometimes referred to as “families of choice” (Dewaele, Cox, Van den Berghe, & Vincke, 2011). It has been argued that traditional conceptions of friendship inadequately describes the range and depth of these relationships (Almack, Seymour, & Bellamy, 2010), with some relationships not easily fitting into a “friend–lover” binary classification system (Roseneil & Budgeon, 2004, p. 138).

Friendships appear to be a greater source of support for older lesbians and gay men compared with their heterosexual counterparts (Weeks, Heaphy, & Donovan, 2003), and serve as a possible protective factor against the effects of homophobia and is linked with greater psychological well-being (Lyons et al., 2013; Masini & Barrett, 2008). For some participants in this study, friendships were very important, particularly for those who had lost partners or who were not in intimate relationships. Participants described how their small social networks provided places where they felt valued, affirmed and safe.

### Ageing, disability and support services

Many participants described the protection and support provided by intimate partners and social networks as being jeopardised with increasing age and disability. As participants aged, many lost partners and friends and found that their friendship networks diminished with few opportunities to meet other older lesbians and gay men. They feared that accessing services for older people would see a return to the institutional control of their youth. Accessing services was conceptualised as stepping outside of existing social networks where they were safe and affirmed and stepping into a heteronormative world where there was little choice but to hide their sexual orientation to escape homophobic abuse from staff and other clients.

To illustrate these potential losses, Patrice described her perspective on accessing a senior citizens group and how she felt she had little in common with the heterosexual people accessing this service. She said:

I've been gay all my life, why am I now at 68 going to a group of people who've been married, have children and join their conversations and their life? I've got nothing in common with them. … We have nothing in common other than the fact we breathe the same air and we eat food and we are human beings. … They've got no concept of my life.

Similarly, Gerri expressed concern that in a retirement village she would have little in common with other residents who would be “looking down their noses because they would be of an age when it was taboo.” Pam also noted that while all older people were vulnerable when accessing services, “you add a different sexuality and it's much, much worse.” Many participants like Cliff believed that there would be few choices other than to go back into the closet.

Given these perspectives it is not surprising that older lesbians and gay men often stepped into the role of caregiver if their partner needed support, rather than access services. At times this support was provided beyond their capacity and at cost to their relationship. Pam, whose partner became seriously ill, described her partner's reluctance to access home services because she was concerned about discrimination. As a consequence, she gave up work to become her partner's full-time caregiver. Pam described how she was a “24/7 carer and it was a big mistake. I think that taking on the carer's role led to the destruction of our relationship. I overdid it.” The change in their relationship dynamic and her partner's health placed a significant burden on their relationship, which later ended.

Intimate relationships were also viewed as a buffer against the challenges of ageing. Larry described how he was able to care for his dying partner and how his partner “was lucky I was here for him … gays die a very, very lonely life.” Where social networks were limited to an intimate relationship, participants felt they had little to live for after their partner died. Some felt isolated and alone, surrounded by services and people who did not understand or value them.

## Discussion

Experiences of relationships appear to be different for older lesbians and gay men compared to their heterosexual counterparts. Many participants in this study described growing up and coming out in a world where institutionalised homophobia was sanctioned and how this led to fractured social supports and a detrimental impact on relationships with families, friends and other forms of social connectedness. Despite these challenges, many found ways to build social networks and to create safe, affirming spaces to enjoy their lives, particularly within their intimate relationships.

Family support has been shown to be a factor in mitigating the effects of discrimination (Masini & Barrett, 2008). However, many of the older lesbians and gay men in this study described having been rejected by family when they disclosed their sexual orientation. While some walked away from families to protect their health, others worked at rebuilding relationships with family over decades. Some scaled back their expectations of family, not expecting full support, and treasured even small gestures of support. On the whole, the frequently negative responses from families appeared to have eroded self-esteem, confidence and trust. They also appeared to influence the ways in which future relationships were negotiated and valued, and how these relationships often became refuges from the hostile world of their youth.

For some older lesbians and gay men, being in intimate relationships was their only source of social support. However, being in an intimate relationship appeared to be a double-edged sword for some participants, with the presence of a partner making sexual orientation more public and increasing the likelihood of discrimination. To avoid discrimination, relationships were often hidden, exacerbating social isolation and placing burden on the relationship. Considerable grief was expressed about the lack of opportunities for recognition of intimate relationships, particularly around the death of a partner. The fact that intimate relationships were seldom acknowledged or celebrated reinforced to some older lesbians and gay men that their sexual orientation was not valued. The many conflicting issues around intimate relationships are perhaps some reasons why friendships were given considerable importance.

However, there was significant divergence in experiences and views relating to heterosexual friendships. Experiences with homophobic heterosexuals resulted in some older lesbians and gay men dismissing the capacity of heterosexuals to genuinely understand and value their sexual orientation. For others, it was about the characteristics of friends, rather than their sexuality. A friend was someone who valued and affirmed their sexuality and the capacity to achieve this was not always considered to be dependent on whether a friend was lesbian, gay or straight. Friendships were negotiated and some were lost when it was apparent that these friends were not accepting of their sexuality. On the whole, participants appeared to be very discerning about who entered and remained in their networks.

The safe and affirming places created by intimate relationships and broader social networks were threatened with increasing age and disability. Over time, partners and friends were lost and social networks diminished. Increasing levels of disability made it difficult to get out and meet other like-minded people. The opportunities to engage with other older lesbians and gay men are also inhibited by geography and by the fact that there are no dedicated spaces in which this engagement can occur (Cronin & King, 2014). Many believed that their safe spaces and affirming networks would be undermined by service providers and other clients who were homophobic and with whom they had nothing in common. A more distressing fear for participants was that they would have to “straighten up” their lives, or hide their sexual orientation if they required residential aged care. Many considered that they would no longer be safe and would lose the possibility of expressing their sexual orientation. Unfortunately, while many services are now embracing strategies to become more inclusive, evidence also shows that older lesbians and gay men receive a lesser standard of care in some services (Barrett, 2008; Barrett et al., 2009; The Equal Rights Center, 2014).

The narratives from this small sample highlight the importance of service providers’ understanding and addressing the needs of older lesbians and gay men. They demonstrate how history and life experiences shape the way that relationships are valued and negotiated and how this potentially affects social networks, health and well-being. They also emphasised the importance of providing culturally safe services, or service providers’ understanding history and institutional discrimination, analysing power imbalances and reflecting on how their own values and beliefs influence service delivery (Nursing Council of New Zealand, 2002).

The study also highlights the need for further research. This study was small, limited to three Australian states, only recruited one transgender person and failed to recruit any bisexual or intersex people. Data was not collected about levels of education, socioeconomic status or other factors that may influence participant's social connectedness, health and well-being. The results cannot be generalised, but rather emphasise that a more substantial body of evidence is required to influence legislation, policy and service delivery. Such research could involve a survey of health and well-being, social connections, caregiving responsibilities, access to services and older people's perspectives on what changes need to occur.

At the time of writing significant reforms occurred in Australia that recognised the social support needs of older lesbians, gay, bisexual, transgender and intersex (LGBTI) people. Reforms include an amendment to the Aged Care Act (Australian Government, 2012) to afford older LGBTI people special needs group status and the development of a National LGBTI Ageing and Aged Care Strategy (Department of Health and Ageing [DoHA], 2012). These reforms have been supported by the allocation of funding for community visiting schemes, or programmes to provide visitors for socially isolated older LGBTI people. Community visitors will be volunteers who value older LGBTI people and have the capacity to build social networks and reduce the fear of social isolation and vulnerability to discrimination. This important reform recognises the challenges to social networks addressed in this paper. The authors hope that further research will identify other strategies to build social networks for older LGBTI people.
